# Detection of Urinary Misfolded Proteins for Imminent Prediction of Preeclampsia in Pregnant Women With Suspected Cases: Protocol for a Prospective Noninterventional Study

**DOI:** 10.2196/54026

**Published:** 2024-04-26

**Authors:** Haiyang Tang, Yijia Tian, Jing Fang, Xiaoying Yuan, Minli Yao, Yujia Wang, Yan Feng, Jia Shu, Yan Ni, Ying Yu, Yuanhe Wang, Ping Liang, Xingmin Li, Xiaoxia Bai

**Affiliations:** 1 Women’s Hospital School of Medicine Zhejiang University Hangzhou China; 2 Department of Obstetrics Lanxi People's Hospital Jinhua China; 3 Shuwen Biotech Co, Ltd Hangzhou China; 4 Department of Obstetrics Ningbo Women and Children's Hospital Ningbo China; 5 Department of Obstetrics Quzhou Maternity and Child Health Care Hospital Quzhou China; 6 Department of Obstetrics Xinchang People's Hospital Shaoxing China; 7 Traditional Chinese Medicine for Reproductive Health Key Laboratory of Zhejiang Province Hangzhou China; 8 Zhejiang Provincial Clinical Research Center for Obstetrics and Gynecology Hangzhou China; 9 Key Laboratory of Women’s Reproductive Health Hangzhou China

**Keywords:** preeclampsia, misfolded protein, congophilia, noninvasive, prospective

## Abstract

**Background:**

Preeclampsia (PE) is one of the most common hypertensive diseases, affecting 2%-8% of all pregnancies. The high maternal and fetal mortality rates of PE are due to a lack of early identification of affected pregnant women that would have led to closer monitoring and care. Recent data suggest that misfolded proteins might be a promising biomarker for PE prediction, which can be detected in urine samples of pregnant women according to their congophilia (aggregated) characteristic.

**Objective:**

The main purpose of this trial is to evaluate the value of the urine congophilia-based detection of misfolded proteins for the imminent prediction of PE in women presenting with suspected PE. The secondary objectives are to demonstrate that the presence of urine misfolded proteins correlates with PE-related maternal or neonatal adverse outcomes, and to establish an accurate PE prediction model by combining misfolded proteins with multiple indicators.

**Methods:**

At least 300 pregnant women with clinical suspicion of PE will be enrolled in this prospective cohort study. Participants should meet the following inclusion criteria in addition to a suspicion of PE: ≥18 years old, gestational week between 20+0 and 33+6, and single pregnancy. Consecutive urine samples will be collected, blinded, and tested for misfolded proteins and other PE-related biomarkers at enrollment and at 4 follow-up visits. Clinical assessments of PE status and related complications for all participants will be performed at regular intervals using strict diagnostic criteria. Investigators and participants will remain blinded to the results. Follow-up will be performed until 42 days postpartum. Data from medical records, including maternal and fetal outcomes, will be collected. The performance of urine misfolded proteins alone and combined with other biomarkers or clinical variables for the prediction of PE will be statistically analyzed.

**Results:**

Enrollment started in July 2023 and was still open upon manuscript submission. As of March 2024, a total of 251 eligible women have been enrolled in the study and enrollment is expected to continue until August 2024. Results analysis is scheduled to start after all participants reach the follow-up endpoint and complete clinical data are collected.

**Conclusions:**

Upon completion of the study, we expect to derive an accurate PE prediction model, which will allow for proactive management of pregnant women with clinical suspicion of PE and possibly reduce the associated adverse pregnancy outcomes. The additional prognostic value of misfolded proteins is also expected to be confirmed.

**Trial Registration:**

Chinese Clinical Trials Registry ChiCTR2300074878; https://www.chictr.org.cn/showproj.html?proj=202096

**International Registered Report Identifier (IRRID):**

PRR1-10.2196/54026

## Introduction

### Background

Preeclampsia (PE) is one of the most common hypertensive diseases in pregnancy, characterized by elevated blood pressure accompanied by proteinuria or organ damage after 20 weeks of gestation [1]. The incidence of PE is approximately 2%-8% worldwide [2,3], and each year, an estimated 76,000 women and 500,000 fetuses die from PE and related complications [4]. Symptoms of PE are not specific, including headache, visual impairment, upper abdominal pain, and shortness of breath, among others. In severe cases, PE may develop into eclampsia, which involves seizures or coma [5-7]. PE may also lead to placental abruption; intrauterine growth restriction; hemolysis with a microangiopathic blood smear, elevated liver enzymes, and low platelets (ie, HELLP syndrome, a life-threatening liver and blood clotting disorder); and other serious health consequences for both the pregnant woman and fetus [8,9]. To date, the treatment of PE has mainly involved the use of antihypertensive medications such as methyldopa, labetalol, and hydralazine to lower blood pressure and prevent further maternal complications [10-12], and magnesium sulfate is also administered to prevent seizures in severe cases [13]. However, the only curative treatment of PE is to deliver the placenta. The current clinical strategy for PE is focused on the early screening and close monitoring of high-risk pregnancies, since low-dose aspirin therapy has proven to be effective in reducing the risk of developing PE by 62% [14]. Although potential biomarkers and ultrasonic techniques for detecting PE have been evaluated in recent decades, none has adequate positive or negative predictive value for the prediction of PE [15-17]. 

With increasing knowledge of the condition, six stages of PE have been proposed [6]. Defective placentation (stage 3) results in oxidative stress, and placental-derived products are released into the maternal blood (stage 4) before PE is diagnosed (stage 5). Recently, an increased level of misfolded proteins in the urine has been identified in patients with PE [18], suggesting that PE might share pathophysiologic features with recognized protein misfolding disorders such as Alzheimer disease. In patients with PE, misfolded proteins such as amyloid beta and transthyretin can accumulate in the circulation to exert neurotoxic effects and activate inflammatory cascades, leading to endothelial dysfunction and oxidative stress. Buhimschi et al [18] demonstrated that a dot test using Congo Red, a synthetic diazo dye with specific affinity for misfolded proteins, to visualize this aggregation feature (ie, congophilia) for detecting the presence of misfolded proteins in urine could serve as a promising diagnostic tool for PE. Li et al [19] subsequently developed a simple and rapid point-of-care testing (POCT) tool to detect the presence of misfolded proteins in urine based on visual observation, which achieved an overall sensitivity of 73.6% that reached up to 83% for severe PE cases.

The traditional approach for PE screening is to identify risk factors based on maternal demographic characteristics and medical history; however, this approach can only identify 35% of total PE cases and 40% of preterm PE cases, with a false-positive rate of approximately 10% [20,21]. A noninvasive laboratory method involving measurement of the ratio of soluble FMS-like tyrosine kinase 1 (sFLT-1) to placental growth factor (PlGF) in the urine proved to be effective in PE diagnosis, achieving a sensitivity of 70%-72.5% with a 5%-14% false-positive rate [22,23]. Thus, combining urine PlGF and sFLT-1 testing with other biomarkers may yield the best predictive performance.

In this trial, we will test a new congophilia-based detection method alone or combined with clinical variables and other urine biomarkers to predict the onset of PE before its clinical manifestation. This work can therefore help clinicians better identify PE earlier, enabling the proactive management and monitoring of pregnant women who exhibit signs or symptoms of PE but do not meet the current diagnostic criteria.

### Objective

The primary objective of this study is to assess the short-term predictive potential of the new congophilia-based detection tool for PE onset in pregnant women with a clinical suspicion of PE. That is, we aim to confirm whether the urine misfolded protein level can be used to rule in or rule out PE in the short term. The secondary objectives are to evaluate whether the urine misfolded protein level correlates with PE-related maternal or neonatal adverse outcomes and to establish an accurate PE prediction model by combining misfolded protein values and multiple indicators.

## Methods

### Study Design

**Figure 1:**
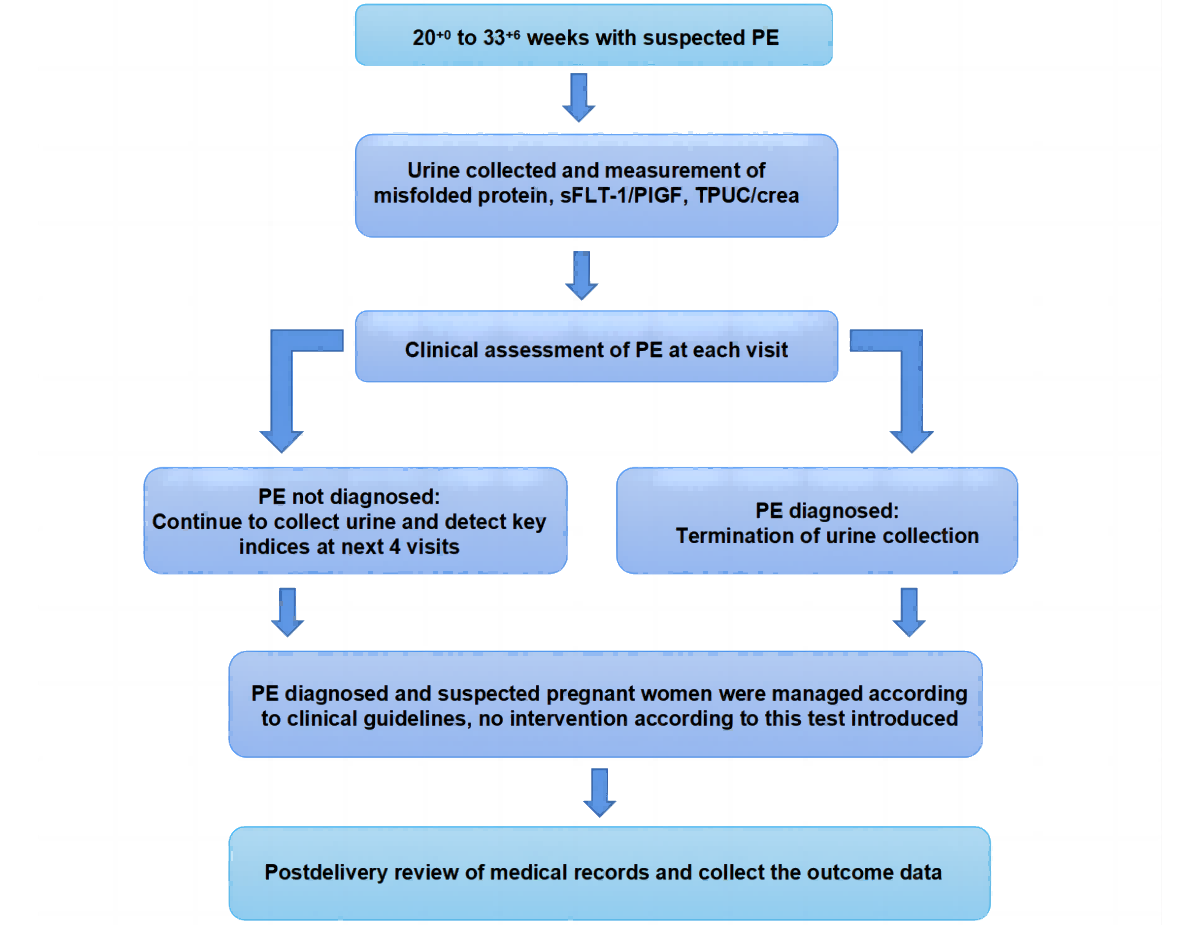
Flowchart of participants with suspected preeclampsia (PE) in the trial. Eligible pregnant women will be enrolled, and consecutive urine samples will be collected at each visit. Urine samples will be blinded and tested for misfolded proteins and other PE-related biomarkers. No intervention according to the test results will be introduced. Required clinical information will be collected from patients’ medical records. crea: creatinine; PlGF: placental growth factor; sFLT-1: soluble FMS-like tyrosine kinase 1; TPUC: Total Protein in Urine.

This is a prospective noninterventional clinical trial planned in accordance with the principles of evidence-based medicine using the PICO (patient, problem, or population; investigated condition; comparison; and outcome) criteria. The study procedure is outlined in Figure 1.

### Ethical Considerations

The trial is being conducted in accordance with ethical principles derived from the Declaration of Helsinki and is in line with Good Clinical Practice and applicable regulatory requirements. The clinical research ethics committee of Women’s Hospital, School of Medicine, Zhejiang University has reviewed the trial protocol, and full ethical approval has been granted (IRB-20230198-K). Each woman identified as eligible for the study is required to give written informed consent, including but not limited to, for primary data collection and secondary analyses of research data prior to inclusion in the trial.

Apart from sample collection and required assessments during follow-up, the study does not involve any further impact on the patients’ clinical procedures. The patients’ PE status will be kept confidential from laboratory technicians, and neither the investigator nor the patient will know the detection results, which ensures that the clinical therapeutic scheme and pregnancy outcomes will not be affected.

### Recruitment

Potential participants will be identified by the research team from the antenatal clinic at Women’s Hospital, School of Medicine, Zhejiang University. Eligible women must meet all inclusion criteria without violating any of the exclusion criteria (Textbox 1) and will be provided with the participant information and consent form to sign. A research team member will provide a verbal explanation of the trial, including a description of the trial processes, the voluntary nature of the trial, and that a decision of whether or not to participate will not affect normal clinical care. No trial-related procedures will be performed on any individual without their prior written informed consent. All participants will be followed up until 42 days postpartum.

**Inclusion and exclusion criteria for determining trial eligibility.**

Inclusion criteria

1. Aged ≥18 years.
2. Gestational week 20+0 to 33+6.
3. Signed written informed consent.
4. Single pregnancy.
5. Clinically suspected preeclampsia (PE), meeting one or more of the following criteria:

(i) new onset of elevated blood pressure (systolic ≥130 mmHg and/or diastolic ≥80 mmHg);

(ii) aggravation of preexisting hypertension (increase of more than 30 mmHg systolic/15 mmHg diastolic from the basic blood pressure in patients without hypertension, or a significant increasing trend or increase beyond the controlled level based on the original treatment for patients with hypertension);

(iii) new onset of proteinuria (no urinary tract infection);

(iv) one or more clinical symptoms indicate suspected PE (other possible causes have been ruled out): upper abdominal pain, headache with impaired vision, fetal growth restriction, thrombocytopenia (platelets count<100×10^9^/L), liver function impairment (elevated blood concentrations of liver transaminases up to twice the normal concentration), and renal impairment (serum creatinine>1.1 mg/dL or a doubling of the serum creatinine concentration in the absence of other renal disease).

Exclusion criteria

1. Confirmed diagnosis of PE/chronic hypertension with PE (superimposed PE).
2. Confirmed diagnosis of eclampsia.
3. Confirmed diagnosis of hemolysis with a microangiopathic blood smear, elevated liver enzymes, and low platelets (HELLP syndrome).
4. Fetal chromosomal abnormalities.
5. Concomitant participation in another clinical study.
6. Received investigational intervention drugs in the past 3 months.
7. Visible hematuria.
8. Preexisting renal disease.

### Sample Size

The target negative predictive value (NPV) is 99% and the target positive predictive value (PPV) is 54.0%. A positive test rate of 15% is assumed. The Type I error is set to .05. A sample size of 280 should be sufficient to detect NPV>90% and PPV>25% with 80% power. Therefore, at least 300 eligible participants will be included in this study considering a dropout rate of 6%. To avoid an overrepresentation of cases of late-onset PE, no more than 50% of the enrolled participants will be at 32 weeks gestation or above and no less than 20% of the enrolled participants will be at gestational week below 28+0 at inclusion.

### Study Groups: Classification of Cases and Controls

Standard diagnostic criteria [24,25] for PE are new onset of hypertension (systolic blood pressure≥140 mmHg or diastolic blood pressure≥90 mmHg measured on two occasions at least 4 hours apart) and new-onset proteinuria (≥300 mg/day or protein/creatinine ratio ≥0.3 mg/dL) after 20 weeks of gestation. Diagnostic criteria in the absence of proteinuria include any of the following: (1) thrombocytopenia (platelets count<100×10^9^/L); (2) liver function impairment (elevated blood concentrations of liver transaminases to twice the normal concentration); (3) renal insufficiency (serum creatinine>1.1 mg/dL or a doubling of the serum creatinine concentration in the absence of other renal disease); (4) pulmonary edema; and (5) central nervous system abnormalities or visual disturbances.

Cases are defined as patients who develop PE at any time between the first visit to 6 weeks (42 days) postpartum.

Controls are patients who do not develop PE throughout the pregnancy and up to 6 weeks (42 days) postpartum.

### Outcome Measures

The primary outcome is the onset of PE after study inclusion. The secondary outcomes are the following maternal and fetal adverse outcomes: maternal death, pulmonary edema, acute renal failure, cerebral hemorrhage, cerebral thrombus, disseminated intravascular coagulation, eclampsia, HELLP syndrome, rupture of placenta, placental abruption, perinatal death, stillbirth, intrauterine growth restriction, small for gestational age, respiratory distress, necrotizing enteritis, intraventricular hemorrhage or subdural and cerebral hemorrhage, neonatal hypoxic encephalopathy, or periventricular leukomalacia. In addition, the date and mode of delivery, fetal weight, and Apgar score are important outcome measures.

### Data Collection

Participant data for this study will be collected by investigators and input into an electronic case report form (eCRF). The data will be verified by the principal investigator when suspicious data entries are noted. The data manager will check the completion of the data for final analysis. The study will include 6 visits plus a postpartum documentation. The first visit starts at enrollment, followed by 4 visits once a week when participants come for their routine prenatal examinations. There will be one visit at delivery as well as one postpartum documentation. On occurrence of pregnancy complications, further visits are possible. The clinical data to be collected during each visit are summarized in Table 1. The time interval between two consecutive visits could be 7±2 days, and adherence to the time schedule is crucial for incorporating participant data into the final analysis.

**Table 1:** Summary of the data collection procedure and timeline.

Data collected	Visit 1	Visit 2	Visit 3	Visit 4	Visit 5	Delivery	Postpartum	Unscheduled visit
Inclusion/exclusion criteria	✓							
Informed consent	✓							
General baseline feature	✓							
Chronic history	✓							
Pregnancy history	✓							
Concomitant medication	✓	✓	✓	✓	✓	✓	✓	✓
BMI	✓	✓	✓	✓	✓			✓
Blood pressure	✓	✓	✓	✓	✓		✓	✓
Sampling and detection	✓	✓	✓	✓	✓			✓
Preeclampsia assessment	✓	✓	✓	✓	✓	✓	✓	✓
Laboratory parameters (blood and urine)	✓	✓	✓	✓	✓			✓
Sonographic data	✓	✓	✓	✓	✓			✓
Delivery outcome						✓		
Pregnant woman status						✓	✓	
Fetal/neonatal status						✓	✓	

### Sampling and Detection

#### Urine Sampling and Storage

All enrolled participants will be provided with a sterile cup to collect no less than 5 milliliters of midstream urine at each visit. The sampled urine tubes will be frozen and stored at –20 °C no later than 4 hours after collection. Samples will be shipped frozen to a local laboratory for further analysis.

#### Urine Coding

At enrollment, each participant will be assigned a specific study number to protect their anonymity. The leading principal investigators will ensure that the participants’ anonymity is maintained in the trial database and during the whole research process. Samples collected from one participant at one time will be labeled with the same number to allow tracking and identification of any aliquot prepared from the original urine and for sample programming in laboratory analyzers. The test results will be held securely and separately from the eCRFs, ensuring that clinical decisions will not be influenced by the test results.

#### Laboratory Detection

Urine samples will be thawed at room temperature and centrifuged at 3000 rpm for 10 minutes. The supernatant will be used for detection.

Urine misfolded proteins will be detected by Pre-Eclampsia Detection Kit (CercaTest RED, Shuwen, China) since misfolded proteins can selectively bind to the Congo Red dye. When a mixture of the Congo Red and urine from a pregnant woman is loaded onto a chromatographic column mounted in a detection cuvette, the presence (positive) or absence (negative) of misfolded proteins can be determined based on the color of the eluent in the cuvette. The eluent will also be applied for quantitative measurement using the HACH DR1900 instrument by detecting the absorbance at a wavelength of 520-600 nanometers.

PlGF and sFLT-1 levels will be detected by the Elecsys PlGF and Elecsys sFlt-1 detection kits (Roche, Germany), respectively. The total urine protein/creatinine ratio will be detected by the Total Protein in Urine/CSF (TPUC) Kit and Creatinine Kit (Mindray, China), all according to the manufacturers’ instructions.

### Selection of Predictor Variables

According to the objective of this study, the misfolded protein level in urine will be considered as a predictor candidate for PE onset or adverse outcomes. Other preset variables include: (1) demographic factors, including high-risk factors of PE; (2) urine biomarkers reported in the literature; (3) medication; and (4) routine clinical variables or laboratory tests in daily practice (Textbox 2). The determination of valid variables will be based on the statistical analysis results with a significance level of *P*<.05.

**Preset variable candidates for model development.**

Demographic factors

- Age
- BMI
- Conception mode
- Parity
- Preexisting hypertension, diabetes, autoimmune diseases (systemic lupus erythematosus, antiphospholipid syndrome)

Urine biomarkers

- Misfolded proteins
- Placental growth factor
- Soluble FMS-like tyrosine kinase 1
- Total protein
- Creatinine

Medication

- Aspirin
- Antihypertensive drugs

Routine clinical variables or laboratory tests

- Blood pressure
- Aspartate aminotransferase
- Lactate dehydrogenase
- Serum creatinine
- Proteinuria (spot urine protein, 24-hour urine protein)
- Uterine artery pulse index

### Statistical Analysis

All variables will be compared between case and control groups. Either parametric or nonparametric tests will be applied for continuous variables and *χ*^2^ tests will be used for categorical variables. Receiver operating characteristic analysis will be used when applicable. Univariable or multivariable logistic regression will be applied to develop the prediction models. All *P* values will be two-tailed. The statistical analysis will be run on SPSS version 27 (IBM) statistical software.

## Results

The study is currently in the patient recruitment phase. Study recruitment started in July 2023. As of March 2024, a total of 251 pregnant women with clinical suspicion of PE have been enrolled in the trial, who will be continuously followed up and monitored for their PE status until 42 days postpartum. Statistical analysis is scheduled to start after all participants reach the follow-up endpoint and complete clinical data are collected.

## Discussion

### Study Implications

Many potential PE biomarkers have been identified using samples from patients with a confirmed diagnosis of PE in comparison with samples obtained from normal pregnancies, which represents a convenient and pragmatic approach for biomarker identification. Although these studies have generated excellent candidate biomarkers for PE detection, there has been less focus on biomarkers that can be used in PE risk prediction. It is possible that biomarkers that are differentially expressed in patients with PE versus those without will fail in a predictive verification study. Thus, a prospective cohort with an uncertain subsequent PE status would provide the best avenue for exploring PE prediction models. To our best knowledge, this is the first prospective study to explore the predictive value of a congophilia-based detection tool in pregnant women with a clinical suspicion of PE in China. The study will be conducted in a high-quality tertiary hospital focused on women’s health. Maternal age of 35 years or older; nulliparity; and preexisting hypertension, diabetes, and autoimmune diseases (eg, systemic lupus erythematosus and antiphospholipid syndrome) are the main high-risk factors for PE [25] and will be included as predictive variables in our analysis. High-risk pregnant women are recommended to use low-dose aspirin to prevent PE; therefore, it is appropriate to consider medication with aspirin as a variable candidate. The crucial biomarkers of PE [22,23], such as PlGF, sFLT-1, and routine clinical variables or laboratory tests, are also under evaluation to build a prediction model. Another important feature of this study is the use of blinding, which ensures that the participants’ laboratory data have no influence on the diagnosis of PE as well as the clinical decisions of treatment.

PE remains one of the most severe pregnancy complications associated with substantial maternal and perinatal morbidity. With current pregnancy trends of advancing age and obesity, the morbidity of PE might increase inevitably in the future. Since the only definitive treatment for PE is to terminate the pregnancy, early screening and continuous monitoring during pregnancy are particularly important for pregnant women with high risk of PE. No effective screening test for PE has been identified. The commonly used clinical screening methods are mostly based on blood samples with precise instruments [26-28], which limits the ability for home self-inspection and timely identification of the disease. Congophilia detection represents a promising new approach for the detection of PE by targeting the misfolded proteins in urine, providing a simple, rapid, and cost-effective method for early diagnosis of this complex condition. This method can be performed using small volumes of urine and no additional instrument is needed, making it suitable for use in resource-limited settings and even as a potential home self-test. Therefore, the affordable, noninvasive, and simple use of this POCT detection tool will enable its wide accessibility for bedside use in a central hospital, low-resource settings, and even at home, which would significantly reduce the serious socioeconomic burden attributed to PE.

### Limitations

Although use of a prospective cohort in undiagnosed populations provides the best avenue for discovering predictive biomarkers, these markers or their combinations must be rigorously validated in internal/external cohorts to ensure their potential for PE prediction. The single-center nature of this study and prospective recruitment may imply the potential for selection bias. In addition, the enrollment was mainly conducted at prenatal clinics, and this might introduce selection bias regarding the severity of disease, as women with sudden onset of organ complications are more often seen at the emergency clinic. Therefore, future investigations with larger populations, along with assessment of the long-term outcomes of infants will be necessary to ensure the accuracy of the prediction model obtained in this trial and to establish the clinical utility of this tool for routine use in pregnancy care.

### Conclusions

This study aims to provide comprehensive evidence on urine misfolded proteins alone or combined with other indicators as a short-term predictive tool for PE. Accurate prediction of PE will allow for proactive management of pregnant women with clinical high risks, and possibly reduce the adverse maternal and fetal outcomes and health care costs associated with hospitalization.

## Abbreviations

eCRF: electronic case report form
HELLP: hemolysis, elevated liver enzymes, and low platelets
NPV: negative predictive value
PE: preeclampsia
PICO: patient, problem, or population; investigated condition; comparison; and outcome
PlGF: placental growth factor
POCT: point-of-care testing
PPV: positive predictive value
sFLT-1: soluble FMS-like tyrosine kinase 1
